# *Tibet Orbivirus*, a Novel *Orbivirus* Species Isolated from *Anopheles maculatus* Mosquitoes in Tibet, China

**DOI:** 10.1371/journal.pone.0088738

**Published:** 2014-02-12

**Authors:** Minghua Li, Yayun Zheng, Guoyan Zhao, Shihong Fu, David Wang, Zhiyu Wang, Guodong Liang

**Affiliations:** 1 State Key Laboratory for Infectious Disease Prevention and Control, Collaborative Innovation Center for Diagnosis and Treatment of Infectious Diseases, National Institute for Viral Disease Control and Prevention, Chinese Center for Disease Control and Prevention, Beijing, China; 2 School of Public Health, Shandong University, Jinan, Shandong Province, China; 3 Washington University, St. Louis, Missouri, United States of America; University of Kansas Medical Center, United States of America

## Abstract

**Background:**

The genus *Orbivirus* includes a number of important pathogenic viruses, including Bluetongue virus (BTV), African horse sickness virus (AHSV), and Epizootic hemorrhagic disease virus (EHDV). In this study we describe the isolation and characterization of an *Orbivirus* strain isolated from *Anopheles maculatus* mosquitoes collected in Tibet, China.

**Methods and Results:**

Initial viral screening identified a viral strain (XZ0906) that caused significant cytopathic effect (CPE) in BHK-21 cells, including rounding, cell rupture, and floating. Although CPE was not observed in insect cells (C6/36), these cells supported viral replication. Polyacrylamide gel analysis revealed a genome consisting of 10 segments of double-stranded RNA (dsRNA), with a distribution pattern of 3-3-3-1. 454 high throughput sequencing of culture supernatant was used for viral identification. Complete genome sequencing was performed by Sanger sequencing in combination with 5′-RACE and 3′-RACE. Sequence analysis demonstrated that all 5′- and 3′- untranslated regions (UTRs) for each of the 10 genome segments contained a series of six highly conserved nucleotides. In addition, homology analysis and phylogenetic analysis based on amino acid sequence was completed, and all results show that virus XZ0906 was not a member of any known species or serotype of Orbivirus, indicating it to be a new species within the genus Orbivirus.

**Conclusions:**

The isolated Orbivirus strain was designated *Tibet Orbivirus*, TIBOV to denote the location from which it was isolated. TIBOV is a novel orbivirus species which is isolated from *Anopheles maculatus* mosquitoes collected in Tibet, China.

## Introduction

There are currently 22 confirmed species of the genus *Orbivirus* in the family *Reoviridae* [1]. This genus includes a number of important pathogenic viruses, including Bluetongue virus (BTV), African horse sickness virus (AHSV), and Epizootic hemorrhagic disease virus (EHDV) [1], [2], which are spread primarily through insect vectors, such as *Culicoides* midges, ticks, mosquitoes, and phlebotomine flies [1], [3]–[6].

Orbiviruses contain a multi-segmented, double-stranded RNA genome, consisting of 10 segments (Seg1–Seg10) of various length, which are identified according to their molecular weight [7]. Partial nucleotide sequences for each of the gene segments for many of the *Orbiviruses* have been published, along with complete genome sequences of some species [3], [5], [8]–[10], allowing for detailed classification and phylogenetic analysis of *Orbiviruses*.

This study describes a viral strain (XZ0906) isolated from *Anopheles maculatus* specimens collected in Tibet, China. All the results of initial viral screening showed a difference between XZ0906 and Yunnan Orbivirus (YUOV), an orbivirus also isolated from China. After whole genome sequencing, amino acid homology and molecular phylogenetic analysis, XZ0906, which is designated as *Tibet Orbivirus* (TIBOV), is identified as a novel species of the genus *Orbivirus*.

## Materials and Methods

### 1. Cell culture

*Aedes albopictus* C6/36 cells and BHK-21 (Baby hamster kidney) cells (ATCC) were used in this study [11], and both cell lines were kept in our laboratory. C6/36 cells were maintained in medium with 45% RMPI 1640 and 45% DMEM (Invitrogen) supplemented with 10% inactive fetal bovine serum (FBS, Invitrogen) and 100 U/mL penicillin and streptomycin. Cells were propagated and maintained at 28°C [11]–[13]. BHK-21 cells were grown in minimal essential medium with Eagle's balanced salt solution supplemented with 10% FBS (Invitrogen), 2 mM glutamine, 0.12% NaHCO_3_, and 100 U/mL penicillin and streptomycin. BHK-21 cells were propagated and maintained at 37°C under a 5% CO_2_ atmosphere [11]–[13].

### 2. Viral isolation

Mosquito samples were collected in Medog County (altitude 1000 m) in the Nyingchi area of Tibet, China during the summer of 2009, and transported to the laboratory in liquid nitrogen containers, following morphological classification and species identification on-site. All specimens were homogenized and centrifuged at 12000×g for 30 min at 4°C. To isolate the virus, 150 µL of supernatant was then added to monolayers of both C6/36 and BHK-21 cells, and cultured at 28 and 37°C, respectively, in a 5% CO_2_ incubator. Cells were monitored at 24-h intervals to identify cytopathic effects (CPE) associated with infection [11]–[13].

### 3. dsRNA-polyacrylamide gel electrophoresis

Viral RNA was isolated as described previously, and analyzed by polyacrylamide gel electrophoresis [13].

### 4. Preparation of viral DNA and RNA and 454 sequencing

Viral DNA was extracted from 200-µL aliquots of virus-infected BHK-21 cell culture supernatants using a QIAamp DNA Blood Mini Kit (Qiagen). Viral RNA was extracted from 140-µL aliquots of virus-infected BHK-21 cell culture supernatant using a QIAamp Viral RNA Mini Kit (Qiagen) according to the manufacturer's instructions. cDNA was made with a Ready-To-Go kit (GE Healthcare) using random hexanucleotide primers. Samples were then amplified as described previously [14], [15]. Amplification products were pooled, adaptor-ligated, and sequenced at the Washington University Genome Sequencing Center on the 454 GS-FLX platform (454 Life Sciences, Branford, CT).

Because the nucleic acids used for sequencing contained a mixture of host cell DNA and viral RNA, sequencing reads were filtered using the custom informatics pipeline VirusHunter [16] to identify viral sequences. Sequences identified as most similar to viruses in the genus *Orbivirus*, as well as those that had no significant hit to any sequence in the GenBank database, were assembled with Newbler (454 Life Sciences) using the default parameters. Sequences were trimmed to remove primer sequences prior to data analysis and assembly.

### 5. Complete genome sequencing including 5′- and 3′-untranslated regions

Reverse-transcription polymerase chain reaction (RT-PCR) was performed to fill in gaps between viral gene sequences obtained with 454 sequencing using contig-specific primers. Total viral RNA was extracted as described in Step 4, cDNA was generated by reverse transcription, and used as a template for complete genome amplification. Next, a set of specific primers was designed to amplify each segment of the viral genome and the amplification products were sequenced using the Sanger method (Table 1). 5′-RACE and 3′-RACE systems (Rapid Amplification of cDNA Ends), Version 2.0 (Invitrogen) were used to amplify the 5′- and 3′-UTRs from each of the 10 segments, respectively. 5′-RACE was performed according to the manufacturer's instructions. For 3′-RACE, a PolyA tail was first added to RNA using a PolyA polymerase. 3′-UTR sequences were then generated by RT-PCR using sequence-specific and oligo-dT-adapter primers. Sequence assembly was performed resulting in a complete viral genome.

**Table 1:** Primers used in this study.

Primer	Sequence (5′-3′)	Position	Orientation
6-1-1F	GTAAAATCACAATGGTCG	1–18	Sense
6-1-1R	TAGCAGCAACTCCCCAAG	826–843	Antisense
6-1-2F	TGGAGGAAGAGGGCGTGAG	679–697	Sense
6-1-2R	TAGAACCCTTTGTTTGGT	1531–1548	Antisense
6-1-3F	AGTCAAGAAAAGGTTTGG	1385–1402	Sense
6-1-3R	CTGAGCGTAAAATAGCGT	2310–2327	Antisense
6-1-4F	ATTTAGCCATGATAGACACG	2152–2171	Sense
6-1-4R	GAGACAATCGCCCTGGTG	3064–3081	Antisense
6-1-5F	ATGCGACCCATACATAAA	2874–2891	Sense
6-1-5R	CTCGTCCTCCGTCACAAC	3786–3803	Antisense
6-1-6F	CTGAAATAATGGATGCGGTTGA	3019–3040	Sense
6-1-6R	GTAAGTGTATCACGGGCGCGCTAAT	3926–3950	Antisense
6-2-1F	GTAAAAACTGACGATGGACGAATTC	1–25	Sense
6-2-1R	CGCATCCGCTCTTGAAAT	940–957	Antisense
6-2-2F	ATTTGAGAAGTGGGAGTT	760–777	Sense
6-2-2R	TTCATGTACGGTGGTAAG	1549–1566	Antisense
6-2-3F	TTATAGATGGTGATTTGCTT	1428–1447	Sense
6-2-3R	CATCCTTACTTCTGACGC	2270–2287	Antisense
6-2-4F	GGGCATACGGCGGAGAAT	2021–2038	Sense
6-2-4R	GTAAGTTTAAACTGTGTGGTGATCG	2864–2888	Antisense
6-3-1F	GTAAAATTTCCGTGGCGATGGCTGA	1–25	Sense
6-3-1R	ACCGCAGGGTTTATAGGT	824–841	Antisense
6-3-2F	GCTCGGACCCACTTTACC	637–654	Sense
6-3-2R	TGCTGCCACAAGCATCAG	1515–1532	Antisense
6-3-3F	TATAATGGATGGGCTGTC	1356–1373	Sense
6-3-3R	GTAGTCTGGCAATCTCGT	2248–2265	Antisense
6-3-4F	TATTGGAGCGTGAAGCAT	2056–2073	Sense
6-3-4R	GTAAGTGTATTCCCGTTGCAGTCGG	2745–2769	Antisense
6-4-1F	GTAAAAACATGCCGGAGCCACATGC	1–25	Sense
6-4-1R	TAGGCGATCCTCAGCAAA	855–872	Antisense
6-4-2F	CGACAGACCAAAAGATAT	734–751	Sense
6-4-2R	TCAACACGTAATCCAATA	1565–1582	Antisense
6-4-3F	TGCAGCGCCTAAAACGAT	986–1003	Sense
6-4-3R	GTAAGTGTAACATGCCTTCCAGATC	1954–1978	Antisense
6-5-1F	GTAAAAAAGTTCTTCGTCGACTGCC	1–25	Sense
6-5-1R	ACCAGCGTCATCGGCATC	955–972	Antisense
6-5-2F	CACCGACAGAAGCAAGGC	789–806	Sense
6-5-2R	GTAAGTGTAAGTTCGATAGAGCGAA	1751–1775	Antisense
6-6-1F	GTAAAAAAGATCGCCTTACGTGCAG	1–25	Sense
6-6-1R	GCTTATCCCCGCAACCAA	915–932	Antisense
6-6-2F	AAGGGATGCAAGAGGAGG	655–672	Sense
6-6-2R	GTAAGTTTAAGATCTAATTACGCTG	1612–1636	Antisense
6-7-1F	GTAAAAATTTGGTGAAGATGGACGC	1–25	Sense
6-7-1R	TCGCTGCTCGCAAACCGT	853–870	Antisense
6-7-2F	GTGGTTGCCTGGAATGGA	681–698	Sense
6-7-2R	GTAAGTGTAATTTGGGAAAACGTAT	1141–1165	Antisense
6-8-1F	GTAAAAAATTCCTAGCAACCATGGA	1–25	Sense
6-8-1R	CCACCTTTGACCACCTTA	866–883	Antisense
6-8-2F	GGTAACCGAGATTCGCTCAA	524–543	Sense
6-8-2R	GTAAGTTTAAATTCCCTCCCCTATA	1118–1142	Antisense
6-9-1F	GTAAAAAATTGCTTATGTCAGCTGC	1–25	Sense
6-9-1R	TGAGCACTACCCACCCTC	565–582	Antisense
6-9-2F	AAGAAGATTCGGTGGTGG	286–303	Sense
6-9-2R	GTAAGTTTTAAATTGCTACGGTCAG	1076–1100	Antisense
6-10-1F	GTAAAAAAGAATGTGGTTGTCATGC	1–25	Sense
6-10-1R	CGATTTGGCCCGTTAGCA	587–604	Antisense
6-10-2F	GATGACGGATGGAATGGC	159–176	Sense
6-10-2R	GTAAGTTGGGTGAATGCGGTGAACT	808–832	Antisense

### 6. Molecular detection of viral genes in cell culture

Viral replication was detected in infected C6/36 and BHK21 cells using RT-PCR for specific regions for TIBOV segment 1 and segment 2. Total RNA was extracted from cell culture supernatants as described in Step 4. cDNA was then generated by reverse transcription, and used as a template for RT-PCR. Gene amplification was performed using primers 6-1-5R and 6-1-5F (primers for Seg1), 6-2-2R and 6-2-2F (primers for Seg2), etc.; detailed sequence information for all primer sequences is shown in Table 1. PCR was performed under the following conditions: one cycle of denaturation at 95°C for 5 min, 35 cycles of 95°C for 1 min (denaturation), 52°C for 1 min (annealing), and 72°C for 1 min (extension), followed by a final extension at 72°C for 10 min. Amplification products were analyzed by gel electrophoresis on a 1% agarose gel.

### 7. Nucleotide and amino acid sequence analysis

Sequences were identified by BLAST analysis (http://www.ncbi.nlm.nih.gov/BLAST/). Multiple sequence alignments were performed using the Clustal X2 software. Phylogenetic analysis of amino acid sequences for each *Orbivirus* gene segment were performed using the MEGA 5.04 software package (www.megasoftware.net). Amino acid sequences were analyzed using PredictProtein (http://www.predictprotein.org/). The background information for all virus strains used in this study is shown in Table 2.

**Table 2:** Information of all virus strains used in this study.

Genus	Species	Abbreviation	Strain/Serotype	GenBank accession no.	
				VP1(RdRp)	T 2
*Genus Orbivirus*	African horsesickness virus	AHSV-1	HS29-62/serotype1	FJ183364	FJ183365
	African horsesickness virus	AHSV-2	HS 02-07/serotype2	FJ196584	FJ196585
	African horsesickness virus	AHSV-4	HS32-62/serotype4	JQ796724	JQ796725
	African horsesickness virus	AHSV-9	E41-02(Or)/serotype9	U94887	DQ868776
	Bluetongue virus	BTV-1	SZ97-1/serotype1	JN848759	JN848760
	Bluetongue virus	BTV-1A	Australia	NA	P20608
	Bluetongue virus	BTV-2	BTV-2IT(L)/serotype2	JN255862	JN255863
	Bluetongue virus	BTV-4	BTV-4IT(L)/serotype4	JN255882	JN255883
	Bluetongue virus	BTV-6	USA2006-01/serotype6	GQ506536	GQ506537
	Bluetongue virus	BTV-9	BTV-9IT(L)/serotype9	JN255902	JN255903
	Bluetongue virus	BTV-12	BTV12-PT2003/serotype12	GU390658	GU390659
	Bluetongue virus	BTV-13	USA	NA	Q65750
	Bluetongue virus	BTV-1S	South Africa	NA	P56582
	Bluetongue virus	BTV-17	USA	NA	P03539
	Changuinola virus	CGLV	BeAr478620	HQ397615	NA
	Corriparta virus	CORV	CSIRO1740	HQ397617	NA
	Corriparta virus	CORV	MRM1	NA	AAM96695
	Epizootic hemorrhagic disease virus	EHDV-1	New Jersey/serotype1	NC_013396	NC_013397
	Epizootic hemorrhagic disease virus	EHDV-2	Ibaraki/serotype2	AM745077	AM745078
	Epizootic hemorrhagic disease virus	EDHV-2	Alberta/serotype2	AM744997	AM744999
	Epizootic hemorrhagic disease virus	EHDV-6	318/serotype6	AM745067	AM745068
	Epizootic hemorrhagic disease virus	EHDV-7	CSIRO 775/serotype7	AM745047	AM745048
	Equine encephalosis virus	EEV	HS103-06	FJ183384	FJ183385
	Eubenangee virus	EUBV	AUS1963/01	JQ070376	JQ070377
	Great Island virus	GIV	CanAr-42	ADM88592	ADM88593
	Broadhaven virus	BRDV	BRDV	NA	P35934
	Kemerovo virus	KEMV	EgAn 1169-61	ADM88609	ADM88610
	Lipovnik virus	LIPV	CzArLip-91	ADM88603	ADM88604
	Tribec virus	TRBV	TRBV	ADM88606	ADM88607
	Itupiranga virus	ITUV	BeAr312086	HQ397639	NA
	Matucare virus	MATV	MARU21343	HQ397640	NA
	Orungo virus	ORUV	IBH11306-84	HQ397641	NA
	Palyam virus	PALV	Chuzan	BAA76549	BAA34936
	St Croix River virus	SCRV	SCRV	AAG34363	AAG34364
	Umatilla virus	UMAV	USA1969/01	AEE98368	AEE98369
	Stretch Lagoon	SLOV	K49460	ACH91290	ACH91291
	Wallal virus	WALV	Ch12048	NA	AAM96693
	Warrego virus	WARV	V5080	ABM92924	ABM92926
	Warrego virus	WARV	Ch9935	AAM96690	AAM96692
	Wongorr virus	WGRV	CSIRO51	HQ397668	NA
	Wongorr virus	WGRV	mrm13443	NA	U56992
	Wongorr virus	WGRV	Paroo-River	NA	U56993
	Wongorr virus	WGRV	V199	NA	U56991
	Yunnan orbivirus	YUOV	YOV-77-2	YP443925	YP443926
	Middle point orbivirus	MPOV	DPP4440	ABU95014	ABU95015
*Genus Phytoreovirus*	Rice dwarf virus	RDV-A	A	BAA14222	NA
	Rice dwarf virus	RDV-Ch	Chinese	AAB18743	NA
	Rice dwarf virus	RDV-H	H	BAA01074	NA
*Genus Rotavirus*	Rotavirus A (Bovine rotavirus A)	BoRV-A/UK	UK WT BRV4A	CAA39085	NA
	Rotavirus A (Bovine rotavirus A)	SiRV-A/SA11	Simian	AAC58684	NA
	Rotavirus C (Porcine rotavirus C)	PoRV-C/Co	Co	AAB00801	NA
*Genus Seadornavirus*	Banna virus	BAV	BAV-Ch	AAF77631	NA
	Kadipiro virus	KDV	JKT-7075	AAF78848	NA
	Liao ning virus	LNV	LNSV-NE9731	AAQ83562	NA
*Genus Cardoreovirus*	Eriocheir sinensis reovirus	ESRV	905	AAT11887	NA
*Genus Mimoreovirus*	Micromonas pusilla reovirus	MPRV	MPRV	AAZ94041	NA
*Genus Aquareovirus*	Aquareovirus A (Chum salmon reovirus)	CSRV	CSRV	AAL31497	NA
	Aquareovirus A(Striped bass reovirus)	SBRV	SBRV	AAM93410	NA
	Aquareovirus C(Grass carp reovirus)	GCRV	GCRV	AAG10436	NA
	Aquareovirus C (Golden shiner reovirus)	GSRV	GSRV	AAM92745	NA
	Aquareovirus G(Golden ide reovirus)	GIRV	GIRV	AAM93415	NA
*Genus Cypovirus*	Dendrlymus punctatus cytoplas-mic polyhedrosis virus-1	DsCPV-1	DsCPV-1	AAN46860	NA
	Lymantria dispar cytoplasmic polyhedrosis virus-14	LdCPV-14	LdCPV-14	AAK73087	NA
*Genus Coltivirus*	Colorado tick fever virus	CTFV	Florio	AAK00595	NA
	Eyach virus	EYAV	Fr578	AAM18342	NA
*GenusDinovernavirus*	Aedes pseudoscutellaris reovirus	APRV	APRV	AAZ94068	NA
*Genus Fijivirus*	Nilaparvata lugens reovirus	NLRV-Iz	Izumo	BAA08542	NA
*Genus Mycoreovirus*	Mycoreovirus 1(Cryphonectria parasitica reovirus)	CpMYRV-1	9B21	AAP45577	NA
	Mycoreovirus 3 (Rosellinia anti-rot virus)	RnMYRV-3	RArV	BAC98431	NA
*Genus Orthoreovirus*	Mammalian orthoreovirus 1	MRV-1	Lang	AAA47234	NA
	Mammalian orthoreovirus 2	MRV-2	Jones	AAA47245	NA
	Mammalian orthoreovirus 3	MRV-3	Dearing	AAA47255	NA
	Mammalian orthoreovirus 4	MRV-4	Ndelle	AAL36027	NA
*Genus Oryzavirus*	Rice ragged stunt virus	RRSV-Th	Thai	AAC36456	NA

**Note:** NA, Not available.

## Results

### 1. Isolation of viral strains

**Figure 1:**
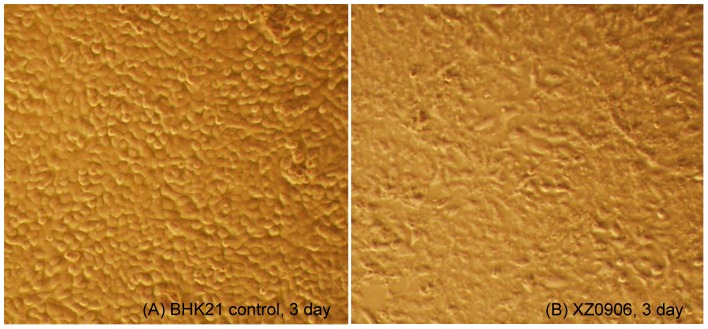
CPE of virus XZ0906 on BHK-21 cells after three days of infection. BHK-21 cells were grown to 80% confluence and inoculated with supernatant harvested from mosquito specimen XZ0906. (A) control BHK21; (B) CPE caused by XZ0906, including rounding, cell rupture.

**Figure 2:**
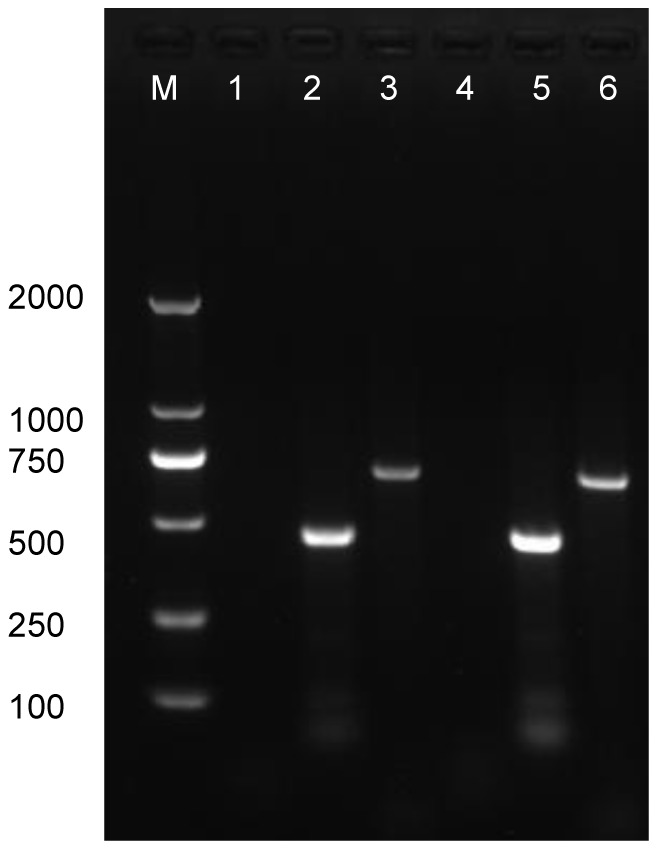
PCR Identification of virus XZ0906 in the culture supernatant of BHK-21 and C6/36 cells. M, Marker DL2000; 1. BHK-21 cell control; 2. BHK-21 cells inoculated with virus XZ0906, the target is an amplicon of 480bp from Segment 1 of XZ0906; 3. BHK-21 cells inoculated with XZ0906, the target is an amplicon of 740bp from Segment 2 of XZ0906; 4. C6/36 cell control; 5. C6/36 cells inoculated with virus XZ0906, the target is an amplicon of 480bp from Segment 1 of XZ0906; 6. C6/36 cells inoculated with virus XZ0906, the target is an amplicon of 740bp from Segment 2 of XZ0906.

*A. maculatus* mosquitoes collected from Tibet, China were homogenized, and the supernatant added to monolayers of C6/36 and BHK-21 cells. Severe CPE was observed in BHK-21 cells three days after inoculation with mosquito lysate XZ0906, characterized by cell rounding, lysis, and floating cells (Figure 1). However, no obvious pathological changes were seen in C6/36 cells cultured with the same mosquito lysate for five days, or after three consecutive passages. Despite the lack of CPE in C6/36 cells, *Orbivirus* Seg1 and Seg2 could be detected by RT-PCR in the supernatant of third-generation C6/36 cultures (Figure 2), indicating that virus XZ0906 could replicate in C6/36 cells.

### 2. Identification of a segmented dsRNA genome

**Figure 3:**
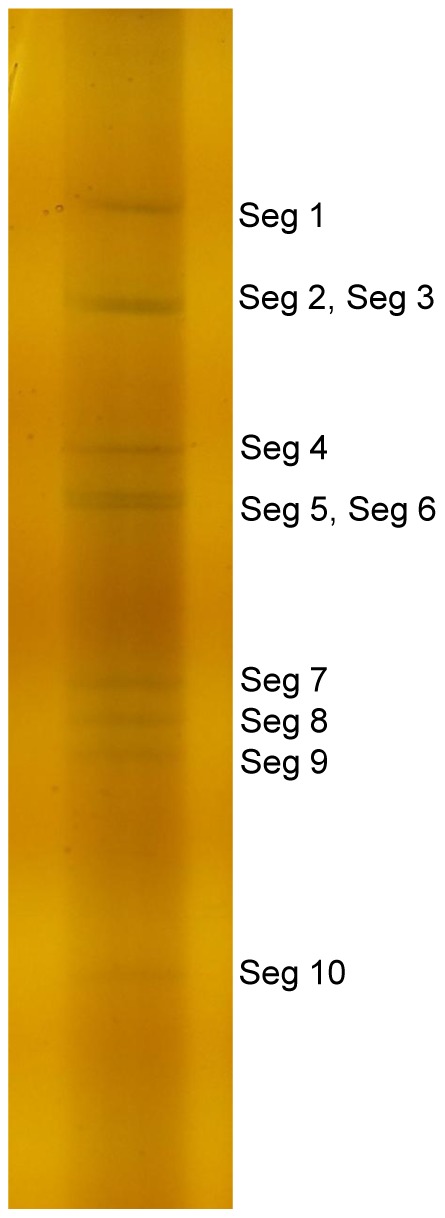
Electrophoretic migration patterns of the dsRNA of virus XZ0906 as determined by polyacrylamide gel electrophoresis. The standard discontinuous polyacrylamide slab gel electrophoresis was used here with a 3.5% acrylamide concentration gel and 10% acrylamide separation gel. After staining with silver nitrate,the genome of XZ0906 was visualized separated into 10 distinct bands.

Viral RNA was harvested from the culture supernatant of infected BHK-21 cells, and analyzed by polyacrylamide gel electrophoresis (PAGE), revealing a genome consisting of 10 dsRNA segments, whose migration pattern was 3-3-3-1 (Figure 3). Within this pattern Seg2 migrated to the same region as Seg3; Seg5 and Seg6 were also difficult to distinguish, indicating that these segments had similar molecular weights. Segments 7, 8, and 9 were also similar in terms of molecular weights, but were easily distinguished from Seg10.

### 3. Preliminary identification of virus XZ0906 using 454 sequencing

Following random PCR amplification, samples were pooled (with barcodes) along with other samples, and sequenced using the Roche/454 FLX Titanium platform, producing a total of 24,929 reads. Sequence data were analyzed using the customized data analysis pipeline VirusHunter [16], identifying 85 unique reads which exhibited 28.1–84.9% sequence identity to viruses in the genus *Orbivirus*.

**Figure 4:**
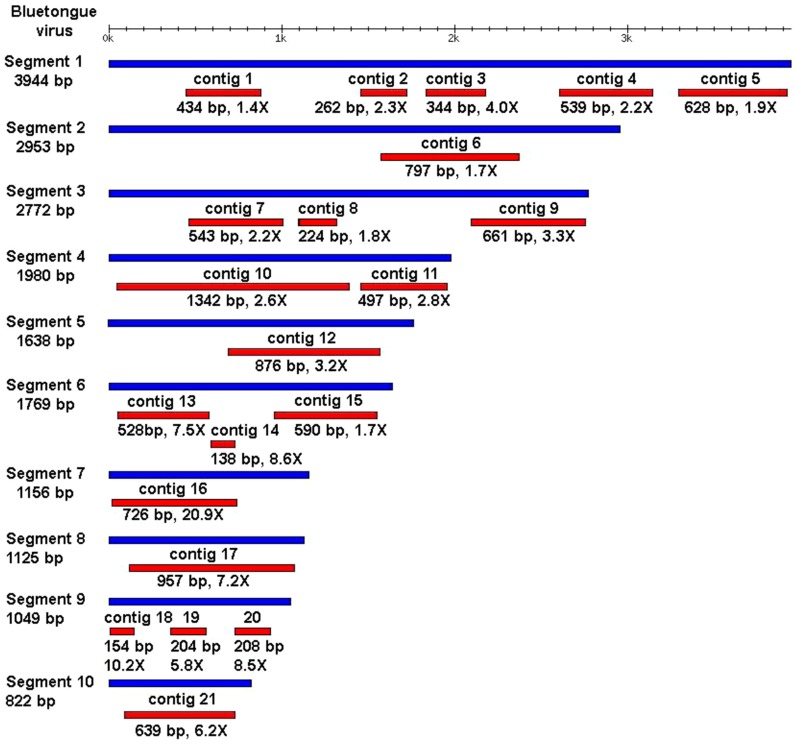
Contigs assembled from 454 sequencing reads compared with BTV. Blue bars represent RNA segments from the BTV reference genome; red bars represent assembled viral contigs. Contig lengths and coverage are shown below each of the respective contigs.

All individual reads with detectable similarity to *Orbivirus*, as well as those sharing no detectable sequence similarity with any sequence in the GenBank database, were used as inputs and assembled into contigs using the Newbler assembler. Twenty-one contigs were assembled, of 138–1342 bp in length, with the greatest similarity to BTV at a coverage depth of 1.4–20.9-fold (Figure 4). Almost-complete RNA sequences were obtained for segments 7, 8, 10. Segments 1, 3, 4, 6 and 9 were represented by two to five contigs; a single contig was identified for segments 2 and 5.

### 4. Sequencing and analysis of virus XZ0906 and other *Orbiviruses*

RT-PCR amplification was used to close the gaps between contigs for each of the 10 segments. Primer walking, together with 5′- and 3′-RACE, were used to sequence the 5′- and 3′-ends of each segment. Finally, Sanger sequencing was employed to confirm sequences using primers newly designed for each of the 10 RNA segments (Table 1); complete sequences for this virus XZ0906 have been deposited in GenBank under accession number(genome segments KF746187 to KF746196).

Sequence analysis identified a stretch of six highly conserved nucleotides present at the ends of the 5′- and 3′-UTRs (5′-GUAAAA and ACUUAC-3′, respectively) for each of 10 gene segments (Table 3).

**Table 3:** Lengths of the coding and untranslated regions of each of the 10 genomic segments of virus XZ0906.

Segment	Length (bp)	Protein (aa)	5′ UTR		3′ UTR	
			Length (bp)	Terminal sequence	Length (bp)	Terminal sequence
S1	3950	1304	11	5′-GUAAAAUC--	24	--ACACUUAC-3′
S2	2888	946	13	5′-GUAAAAAC--	34	--AAACUUAC-3′
S3	2769	899	17	5′-GUAAAAUU--	52	--ACACUUAC-3′
S4	1978	643	8	5′-GUAAAAAC--	38	--ACACUUAC-3′
S5	1775	554	31	5′-GUAAAAAA--	79	--ACACUUAC-3′
S6	1636	526	26	5′-GUAAAAAA--	29	--AAACUUAC-3′
S7	1165	349	17	5′-GUAAAAAU--	98	--ACACUUAC-3′
S8	1142	359	20	5′-GUAAAAAA--	42	--AAACUUAC-3′
S9	1100	346	14	5′-GUAAAAAA--	45	--AAACUUAC-3′
S10	832	234	21	5′-GUAAAAAA--	106	--CAACUUAC-3′

Significant differences were observed in both the nucleotide and amino acid sequences of virus XZ0906 relative to other members of the genus Orbivirus (Table 4). The VP1 protein (RNA-dependent RNA polymerase, RdRp), encoded by Seg1, shared 35.3% (SCRV)-72.9% (EHDV-6) identity at the amino acid level to the six selected Orbiviruses. Protein T2, encoded by Seg3 of XZ0906, shared 22.9% (SCRV) to 75.9% (BTV-6) identity (Table 4).

**Table 4:** Comparison of each segment between virus XZ0906 and other Orbiviruses in nucleotide numbers and amino acid identities.

Segment	AHSV-4		BTV-6		EHDV-6		PALV		SCRV		YUOV	
	nt	aa(%)	nt	aa(%)	nt	aa(%)	nt	aa(%)	nt	aa(%)	nt	aa(%)
S1	3965	1305(59.8)	3944	1302(71.9)	3942	1302(72.9)	3930	1295(59.2)	4089	1345(35.3)	3993	1315(47.8)
S2	3229	1060(9.9)	2922	955(28.8)	2971	972(24.6)	3055	1002(15.6)	2747	890(16.7)	2900	940(16.3)
S3	2792	905(58.5)	2772	901(75.9)	2768	899(75.8)	2774	904(58.0)	2024	654(13.1)	2688	873(8.8)
S4	1978	642(50.5)	1981	644(65.5)	1983	644(64.4)	1967	640(48.7)	2017	643(34.2)	1993	645(40.7)
S5	1748	548(27.6)	1769	552(38.5)	1803	551(41.6)	1764	545(25.3)	1664	517(8.8)	1957	574(20.1)
S6	1566	505(43.6)	1637	526(58.4)	1641	527(61.4)	1610	521(43.3)	1657	517(8.6)	1683	535(31.6)
S7	1167	349(56.7)	1157	349(69.1)	1162	349(69.3)	1151	348(54.1)	1463	462(8.8)	1504	435(17.2)
S8	1166	365(36.3)	1125	354(47.3)	1186	373(44.5)	1059	333(40.3)	1256	379(9.9)	1191	355(16.4)
S9	1160	366(32.9)	1046	328(52.4)	1140	359(46.5)	877	272(43.3)	764	232(35.3)	1082	338(39.8)
S10	756	217(30.7)	822	229(53.9)	810	228(51.0)	728	211(28.0)	764	224(17.4)	825	253(14.9)

**Note:** As the T2 protein of Orbiviruses had important functions in virus protein/RNA structure and assembly, amino acid homology analysis for the T2 protein of TIBOV (T2 = VP3) compared to the T2 proteins of the above mentioned orbiviruses is presented:

AHSV-4(T2 = VP3):58.5%; BTV-6(T2 = VP3):75.9%; EHDV-6(T2 = VP3):75.8%; PALV(T2 = VP3):58.0%;

SCRV(T2 = VP2):22.9%; YUOV(T2 = VP2):37.6%.

### 5. Phylogenetic analysis and classification of virus XZ0906

#### 5.1. Phylogenetic analysis of virus XZ0906 based on VP1 amino acid sequences

**Figure 5:**
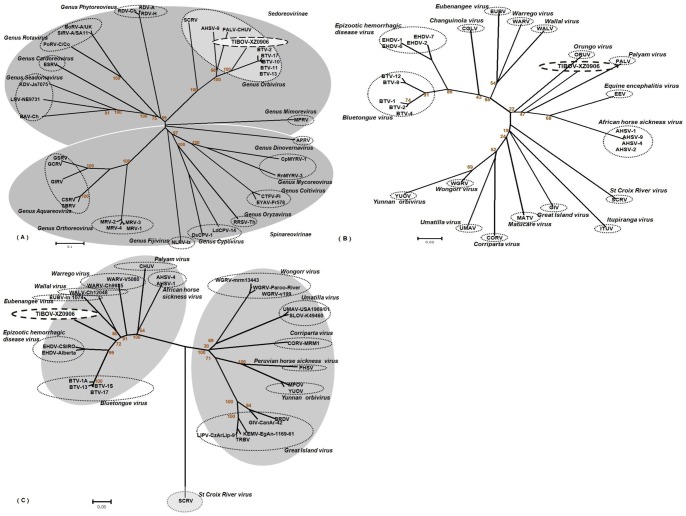
Phylogenetic analysis of VP1 amino acid sequences from (A) *Reoviridae* and (B) *Orbivirus* strains. (C) Phylogenetic analysis of T2 amino acid sequences from 29 *Orbivirus* strains. These analysis employed a neighbor-joining method (using the P-distance algorithm) using the MEGA version 5.04 software package (www.megasoftware.net). Bootstrap probabilities for each node were calculated using 1000 replicates. Scale bars indicate the number of amino acids substitutions per site. In Figure 5(C), as many of the available sequences are incomplete, analysis is based on partial sequences (residues 356-567 relative to the BTV-1A sequence). Abbreviations and serotypes (or strain name) are shown in the radial tree image of Figure 5. GenBank accession numbers and further details of the sequences can be found in Table 2.

To better understand the taxonomic classification of virus XZ0906, the amino acid sequences of 37 VP1 proteins (Table 2) covering 14 genera within the family *Reoviridae* were obtained from GenBank, and used to construct a phylogenetic tree. These 37 virus strains (including different species and different serotype of one species) readily clustered into 14 evolutionary branches, with virus XZ0906 clustering within the genus *Orbivirus* branch (Figure 5(A)). To further establish the taxonomic classification of virus XZ0906, VP1 amino acid sequences from 28 known *Orbivirus* strains were used to construct a phylogenetic tree specific to this genus (Table 2). This analysis shows that virus XZ0906 forms a unique phylogenetic branch independent of any known *Orbivirus* species (Figure 5(B)).

#### 5.2. Phylogenetic analysis based on the T2 protein amino acid sequence

The amino acid sequence of the T2 protein is an important marker used to classify species within the genus *Orbivirus*. T2 amino acid sequences from 29 known *Orbivirus* strains, along with the equivalent region from virus XZ0906, were selected to construct a phylogenetic tree. This analysis showed that virus XZ0906 is independent of any known *Orbivirus* species (Figure 5(C)). From these results, we determined virus XZ0906 to represent a novel species within genus *Orbivirus*. This novel species was given the name *Tibet Orbivirus,* TIBOV to reflect the location from which it was isolated.

## Discussion

According to the 9^th^ meeting report of the International Committee on the Taxonomy of Viruses (ICTV), the *Reoviridae* family consists of 15 genera: *Orbivirus*, *Rotavirus*, *Seadornavirus*, *Phytoreovirus*, *Cardoreovirus*, *Mimoreovirus*, *Aquareovirus*, *Coltivirus*, *Cypovirus*, *Dinovernavirus*, *Fijivirus*, *Idnoreovirus*, *Mycoreovirus Orthoreovirus, and Oryzavirus* [1]. All *Reoviridae* genomes consist of multi-segmented dsRNA; for example, the genome of *Seadornavirus*, *Rotavirus*, and *Orbivirus* contain 12, 11, and 10 dsRNA segments, respectively [2], [10], [17], [18]. Here we describe a novel orbivirus species isolated from mosquitoes collected in Tibet. This virus has many features characteristic of orbiviruses.

UTRs were detected at both the 5′ and 3′-ends of all 10 TIBOV gene segments. The lengths of these UTRs were highly variable; however, all 3′-UTRs contained a stretch of six highly conserved nucleotides at the end, which is a defining molecular characteristic used in the identification of Orbiviruses [8]. For BTV, AHSV, PALV, and Equine encephalosis virus (EEV), this stretch of six conserved nucleotides is readily detected in the 3′-UTRs of each gene segment [1], [4]; however, no such sequences are found at their corresponding 5′-ends. Among the 10 gene segments in Yunnan virus (YUOV), a recently identified *Orbivirus* isolated from mosquitoes in Yunnan, China, nine (Seg2–Seg10) contained a conserved seven-nucleotide sequence at the 5′-UTR end, but only three conserved nucleotide sequences at the 3′-end [4]. Among the 10 gene segments of *Tibet Orbivirus*, TIBOV, six conserved nucleotide sequences were detected in both end of the 5′- and 3′-UTRs (5′-GUAAAA and ACUUAC-3′, respectively); these sequences were distinct from those in any other *Orbivirus* species.

The *Orbivirus* RNA-dependent RNA polymerases (RdRp), which is encoded by the Seg1 gene (VP1), is an important marker for species identification [4], [8]. The VP1 protein sequence similarities of TIBOV to those of other *Orbivirus* species were 35.3–72.9% (Table 4), indicating that TIBOV constituted a novel member of the genus *Orbiviruses*. In addition, the T2 protein of *Orbivirus* is used to classify serotypes within the genus, with a threshold >91% identity at the amino acid level [4], [19], [20]. Such as Middle Point orbivirus (MPOV), which is isolated from Australian bovine serum specimens in 1998, exhibited up to 99% identity with YUOV, indicating that MPOV and YUOV were different serotypes of the same virus species [21]. The T2 protein from TIBOV shared 22.9–75.9% amino acid identity with those from six representative *Orbiviruses*, including YUOV (37.6%), well below the 91% threshold (Table 4). Together with phylogenetic analysis of both VP1(Figure 5(A),5(B)) and T2 (Figure 5(C)) amino acid sequences, we can draw a conclusion that TIBOV represented a new species within the genus *Orbivirus*, rather than a serotype of a previously described *Orbivirus*.

As previously described, YUOV is a new species of *Orbivirus* isolated from *Culex tritaeniorhynchus* specimens collected in Yunnan, China [4]. The genome of this virus consists of 10 dsRNA segments, and exhibits a 3-4-2-1 migration pattern when resolved in an agarose gel. Preliminary analysis of this virus showed clear CPE in *Aedes albopictus* cells, but no CPE or viral replication in mammalian cells (BHK and Vero cells) [4]. TIBOV isolated from mosquito specimens collected in Tibet in this study represents the second *Orbivirus* isolated from mosquito specimens in China. Substantial differences between YUOV and TIBOV were evident, including replication and toxicity to insect and mammalian cells, migration profiles, protein sequences, and the presence of conserved nucleotide sequences in the 5′-UTR and 3′-UTRs. Together, these results demonstrate that TIBOV is distinct from YUOV, and highlights the level of genetic diversity within *Orbiviruses* in China.

*Orbiviruses* can be transmitted by ticks or other hematophagous insect-vectors, including *Culicoides*, mosquitoes, and sand flies [1], [9]. The phylogenetic analyses (Figure 5(C)) indicated that TIBOV, isolated from *A. maculatus*, clustered with *Orbiviruses* which are transmitted primarily by *Culicoides* [1], [4], [9], [10], such as BTV, EHDV, and AHSV. TIBOV is more distantly related to *Orbiviruses* which are isolated from mosquito specimens, such as YUOV [3], Peruvian horse sickness virus (PHSV) [9], Umatilla virus (UMAV) [10], and Stretch Lagoon *Orbivirus* (SLOV) [9], [10]. Further study is necessary to determine if TIBOV is transmitted exclusively through *A. maculatus*, or can be spread by other blood-sucking insects.

TIBOV was isolated from *A. maculatus* specimens collected at a pigsty in rural Tibet. It is currently unknown whether TIBOV can infect either humans or animals. In order to determine whether this virus poses a risk to public health, serological studies to define potential human and animal exposures to TIBOV are needed.
